# Species H Rotavirus Detected in Piglets with Diarrhea, Brazil, 2012

**DOI:** 10.3201/eid2006.130776

**Published:** 2014-06

**Authors:** Bruna L.D. Molinari, Elis Lorenzetti, Rodrigo A.A. Otonel, Alice F. Alfieri, Amauri A. Alfieri

**Affiliations:** UniversidadeEstadual de Londrina, Londrina, Parana, Brazil

**Keywords:** Pigs, piglets, diarrhea, viruses, group H rotavirus, species H rotavirus, *Reoviridae*, porcine rotavirus, Brazil

## Abstract

We determined nucleotide and deduced amino acid sequences of the rotavirus gene encoding viral protein 6 from 3 fecal samples collected from piglets with diarrhea in Brazil, 2012. The analyses showed that the porcine rotavirus strains in Brazil are closely related to the novel species H rotavirus.

Rotaviruses (RVs) form a genus of the family *Reoviridae* and are a common cause of viral gastroenteritis in humans and animals (*1*). The RV genome consists of 11 segments of double-stranded RNA that encode 6 structural viral protein (VP1–4, VP6, and VP7) and 6 nonstructural proteins (NSP1–6) (*1*). RVs have been classified into 7 species, which are also known as groups, termed A–G, on the basis of the antigenicity and genetic characteristics of VP6 (*2*,*3*). *Rotavirus* A (RVA) infections cause severe diarrhea in infants and young children worldwide but can also infect adults, other mammals, and birds (*1*). *Rotavirus* B (RVB) infections were first associated with severe diarrhea in adults (*4*) and have also been detected in cattle, pigs, sheep, and rats (*5*–*7*).

In addition to the 7 established species of RV, Matthijnssens et al. (*2*) recently proposed the new *Rotavirus* H (RVH) on the basis of VP6 sequence analysis. This species includes the following: the novel adult diarrhea RV strain (ADRV-N) isolated from specimens collected during an outbreak of gastroenteritis among adults during 1997 in China (*8*); strain J19, identified during the same outbreak in China in 1997 (*9*); the human *Rotavirus* B219, detected in a sporadic case of diarrhea in Bangladesh during 2002 (*10*,*11*); and the porcine RV strain SKA-1 that was isolated from a pig with diarrhea in Japan (*12*).

In this study, we determined the VP6 nucleotide sequence for 3 RV-positive fecal samples obtained from piglets with diarrhea in Brazil during 2012. A comparative analysis with other VP6 genes showed that the porcine RV strain from Brazil is closely related to the novel RVH.

A molecular study of RVB infection on a pig farm in Mato Grosso do Sul in the Central-West region of Brazil was performed during an outbreak of diarrheal illness in 2012. A total of 59 diarrheic fecal specimens were collected from 59 piglets whose ages ranged from 12 to 35 days, and the presence of RV was investigated by using polyacrylamide gel electrophoresis (*13*). Eight samples that showed RVB dsRNA pattern (4:2:2:3) and 2 that showed polyacrylamide gel electrophoresis inconclusive results were subjected to reverse transcription PCR (RT-PCR) by using the primer pair described by Gouvea et al. (*14*), which were designed to amplify a partial fragment of the NSP2 gene of RVB. All 10 samples were positive for RVB on the basis of the amplification of the 434-bp target fragment. To amplify larger fragments of the distinctive RVB NSP2 gene, we submitted the 10 fecal samples to RT-PCR using the primer pairs NSP2–1 F/R (993 bp), and NSP2–2 F/R (938 bp) as described by Suzuki et al. (*15*). Products of 993 bp expected for amplification reaction with a NSP2–1 primer pair were obtained for 7 of the samples. The remaining 3 samples (BR59, BR60, and BR63), from 35-day-old piglets, did not generate the expected fragments with any of the primer pairs. However, because of an unexpected annealing of the NSP2–2 primer pair in RT-PCR, shorter (≈750 bp) amplicons were generated for these 3 samples.

The nonspecific amplification products of the 3 samples were purified and sequenced with NSP2–2 forward and reverse primers by using the BigDye Terminator v3.1 Cycle Sequencing Reaction Kit (Applied Biosystems, Foster City, CA, USA) on an automated sequencer (ABI3500). Similarity searches were performed with sequences deposited in GenBank by using BLAST software (http://blast.ncbi.nlm.nih.gov/Blast.cgi?CMD=Web&PAGE_TYPE=BlastHome). Of note, the highest nucleotide identities were obtained for the VP4 genes of the RVH strains SKA-1 (89%), B219 (72%), and J19 (70%). The VP4 nucleotide sequence alignment of the RVH SKA-1 strain and the nonspecific product was performed from the nucleotide positions 1792–2433 by using MEGA (v. 6) (http://www.megasoftware.net/).

To confirm the similarity of the samples BR59, BR60, and BR63 with RVH, we performed an additional set of RT-PCRs using 2 new primer pairs designed on the basis of the complete sequence of the VP6 gene of the porcine RVH strain SKA-1 (*12*) (Table 1). Phylogenetic analysis and nucleotide distance calculations were performed by using MEGA (v. 6) and BioEdit (v. 7.0.8.0) software (http://www.mbio.ncsu.edu/bioedit/bioedit.html).

**Table 1:** Oligonucleotide primers designed from the VP6 gene of the SKA-1 rotavirus strain for reverse transcription PCR sequence analysis, Brazil, 2012

Primer	Sequence, 5′→3′	Nucleotide position
VP6/RVN-1F	TGCTACAAGTGACCCACAAGG	11–31
VP6/RVN-1R	GCCATCTTTCCAGTGGCTCT	581–600
VP6/RVN-2F	ACCAGGTGGAGCAACAAACA	529–548
VP6/RVN-2R	CAGTGCGTGACCAGATCTCA	1225–1244

VP, viral protein.

The pairwise comparisons of the VP6 nucleotide and inferred amino acid sequences of the 3 specimens revealed 100% nt and aa identities among them. In contrast, the BR59, BR60, and BR63 sequences showed <36% nt identity (<13.5% aa identity) with cogent sequences of *Rotavirus* A, C, D, and F, and 49.7%–51.6% nt identity (35.8%–38.3% aa identity), respectively, with RVB and RVG. The specimens had relatively high identities with RVH (71.7%–85.5% and 75.7%–96.9% at the nt and aa levels, respectively). The highest identity was shared with the VP6 gene of the porcine RVH SKA-1 strain. (Table 2) The phylogenetic tree (Figure) inferred from the VP6 sequences was separated into distinct phylogenetic clusters representative of RV species. The BR59, BR60, and BR63 samples grouped closest to the RVH species. Although they segregated in a different branch, they clearly were within the RVH cluster.

**Table 2:** Identities of nucleotide and amino acid VP6 gene sequences (nt 24–1221) of the porcine RV samples BR59, BR60, and BR63, compared with the VP6 sequences from representative RV strains of different species, Brazil, 2012*†

Species	Rotavirus	
	Strain (species origin)	% Identity, nt (aa)
A	KU (human)	35.6 (11)
	UK (bovine)	35.2 (11.2)
	OSU (porcine)	34.7 (11.7)
B	ADRV (human)	51.4 (35.8)
	CAL-1 (human)	50.7 (36.8)
	Bang 373 (human)	51 (36.3)
	DB176 (bovine)	49.7 (37.9)
	IDIR (murine)	50.2 (37.4)
C	Bristol (human)	35 (8.7)
	Toyama (bovine)	34.5 (9)
	Cowden (porcine)	34.7 (8.5)
D	05V0049 (chicken)	35.2 (13.2)
F	03V0568 (chicken)	35.5 (10)
G	03V0567 (chicken)	51.6 (38.3)
H	ADRV-N (human)	72.3 (76.4)
	B219 (human)	71.7 (76.2)
	J19 (human)	72.3 (75.7)
	SKA-1 (porcine)	85.5 (96.9)

*VP, viral protein; RV, rotavirus.  †The exact position of the 1,197 fragments of the porcine samples BR59, BR60, and BR63 VP6 gene entered into the phylogenetic comparison was based on the RVH SKA-1 VP6 complete gene. GenBank accession nos.: BR59 (KF021619), BR60 (KF021620), BR63 (KF021621**)**, KU (AB022768), UK (X53667), OSU (AF317123), ADRV (M55982), CAL-1 (AB037931), Bang 373 (AY238389), DB176 (GQ358713), IDIR (M84456), Bristol (X59843), Toyama (AB738416), Cowden (M94157), 05V0049 (GU733448), 03V0568 (HQ403603), 03V0567 (HQ403604), ADRV-N (AY632080), B219 (DQ168033), J19 (DQ113902), and SKA-1 (AB576626).

**Figure:**
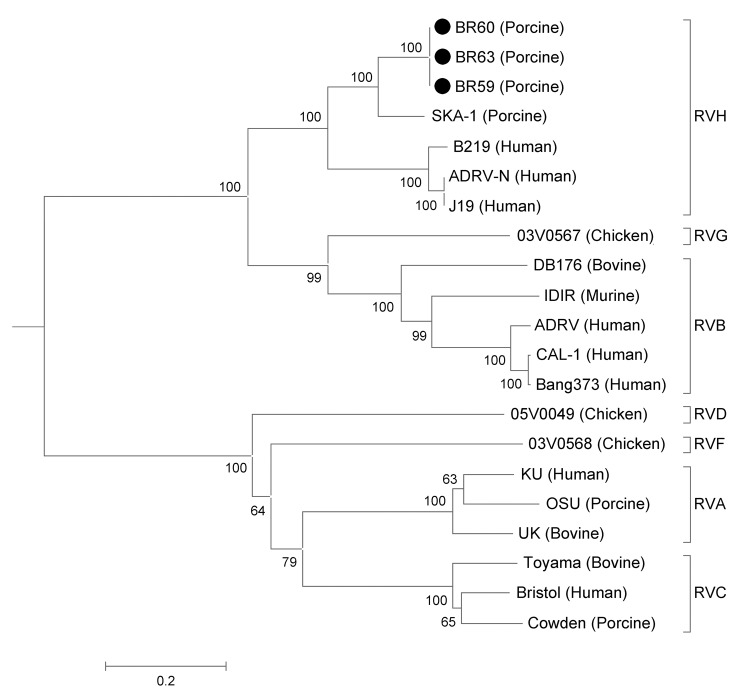
Phylogenetic tree showing the inferred evolutionary relationships among representative rotavirus (RV) strains belonging to species A, B, C, D, F, G, and H, as well as the samples BR59, BR60, and BR63 based on an 1,197-bp fragment of the viral protein 6 (VP6) gene. The tree was constructed by using the neighbor-joining method and the Kimura 2-parameter nucleotide substitution model. Bootstrapping was statistically supported with 1,000 replicates. Scale bar indicates nucleotide substitutions per site. The VP6 gene sequences of the following strains were obtained from the GenBank database (accession nos**.):** BR59 (KF021619), BR60 (KF021620), BR63 (KF021621**)**, KU (AB022768), UK (X53667), OSU (AF317123), ADRV (M55982), CAL-1 (AB037931), Bang373 (AY238389), DB176 (GQ358713), IDIR (M84456), Bristol (X59843), Toyama (AB738416), Cowden (M94157), 05V0049 (GU733448), 03V0568 (HQ403603), 03V0567 (HQ403604), ADRV-N (AY632080), B219 (DQ168033), J19 (DQ113902), and SKA-1 (AB576626).

## Conclusions

The 3 rotavirus samples, BR59, BR60, and BR63, detected in diarrheic fecal specimens from 35-day-old piglets from the same farm shared 100% nt and aa identities of their VP6 gene sequences, suggesting that the specimens represented the same local rotavirus strain. Although initial RT-PCR results by using RVB-specific NSP2 primers suggested that these samples were RVB, sequence analysis of the VP6 gene showed that they are different from RVB.

Matthijnssens et al. (*2*) proposed a 53%-aa cutoff value for VP6 to be used for the differentiation of distinct RV species. The 3 rotavirus samples included in this study showed a VP6 aa identity ranging 76.1% (human strain) to 96.9% (porcine strain) when compared with RVH and are thus considered to belong to the novel species RVH. Prior to this study, 1 porcine RVH strain (SKA-1), detected in Japan, had been reported (*2*,*12*).

The VP6 nucleotide and amino acid sequences of BR59, BR60, and BR63 samples did not show high identities with VP6 sequences from RVs A, C, D, and F, but showed a moderate level of relatedness to VP6 sequences of species RVB, in agreement with previous reports (*10*,*12*). Of note, BR59, BR60, and BR63 sequences shared high similarities with an RVG strain found in chickens, in both nt (51.6%) and aa (38.3%) levels, compared with RVB. This similarity is also evident in the phylogenetic tree, in which these 3 samples cluster closer to RVG and RVB than to the other species of RV.

Our study reports the detection of a porcine RVH from the Americas. One porcine strain, SKA-1, was previously isolated in Japan; there have been no other reports of porcine RVH. Very little information is available regarding this new RV species, strains of which infect humans and piglets. To date, strains of RVH have been detected in China, Bangladesh, Japan, and now in Brazil. The scarcity of molecular and epidemiologic information on these viruses results from lack of appropriate diagnostic tools. Extensive epidemiologic studies are needed to determine the worldwide dissemination and prevalence of this rotavirus species and its effects on diarrheal diseases.
